# Rethinking the seven-day treatment-free interval in T-cell engager therapy using agent-based modeling

**DOI:** 10.1101/2025.11.17.688873

**Published:** 2025-11-17

**Authors:** Nina Obertopp, Matthew Froid, Shari Pilon-Thomas, David Basanta

**Affiliations:** 1 Cancer Biology Ph.D. Program, University of South Florida, Tampa, Florida, USA; 2 Department of Immunology, H. Lee Moffitt Cancer Center & Research Institute, Tampa, Florida, USA; 3 Department of Integrated Mathematical Oncology, H. Lee Moffitt Cancer Center & Research Institute, Tampa, Florida, USA

## Abstract

**Background:**

The CD3/CD19 bispecific T cell engager (TCE) blinatumomab has shown efficacy in relapsed/refractory (R/R) B-cell acute lymphoblastic leukemia (B-ALL), but response rates are often limited by T cell exhaustion. Recent preclinical studies suggest that incorporating treatment-free intervals (TFIs) into dosing schedules may enhance therapeutic outcomes.

**Methods:**

To systematically evaluate alternative TFI strategies, we developed an agent-based model (ABM) of tumor–T cell interactions under various blinatumomab dosing regimens. The model was calibrated using published *in vitro* data and incorporated spatial, stochastic, and mechanistic rules governing T cell activation, cytotoxicity, proliferation, and exhaustion.

**Results:**

Our ABM recapitulates experimental observations showing that a 7-day TFI improved T cell function over continuous dosing during the initial 28-day treatment period. However, when simulations were extended to a full 42-day cycle to mimic clinical regimen, this advantage was lost. In contrast, shorter TFIs consistently outperformed both 7-day and continuous schedules, leading to superior tumor control at all timepoints. A translationally oriented Monday-through-Friday (MO_FR) regimen also achieved comparable benefits.

**Conclusions:**

Our results indicate that the empirically tested 7-day TFI schedule may not be optimal. TFI with shorter intervals as well as translationally relevant schedules such as MO_FR, may offer greater therapeutic benefit. This work demonstrates the value of ABM in preclinical immunotherapy design and supports model-guided refinement of TCE dosing strategies prior to clinical translation. Future work will focus on validating these predictions in more complex *in vivo* models and leveraging patient-derived data to guide personalized TCE treatment design.

## Introduction

Acute lymphoblastic leukemia (ALL) is the most common childhood cancer; adults account for just under half of cases.^1^ While frontline chemotherapy induces long-term remission in approximately 80–85% of children and 40–50% of adults with newly diagnosed ALL, outcomes remain poor for patients with relapsed or refractory (R/R) disease.^2^ The CD3/CD19-directed bispecific T cell engager (TCE) blinatumomab, FDA-approved since 2014, has shown substantial activity in both adult and pediatric patients with relapsed or refractory B-cell ALL (R/R B-ALL). In R/R B-ALL, blinatumomab induces complete remission in up to 69% of patients.^3^ While blinatumomab has reshaped treatment options and is increasingly incorporated into frontline regimens, resistance, relapse, and modest long-term survival in some patient subgroups highlight the need to better understand and overcome mechanisms of treatment failure.^2^

Blinatumomab is administered according to disease status and patient weight, with adults with R/R B-ALL typically receiving multiple 42-day treatment cycles. Each cycle consists of 28 days of continuous intravenous infusion followed by a 14-day resting phase.^4^ Importantly, the treatment holiday was first included in the original 6-week trial schema as a safety window for adverse-event monitoring, recovery, and infusion logistics and was retained in labeling after regulators observed that peripheral B-cell suppression persisted during the break.^5^ Philipp et al. evaluated the initial 28 days of a treatment cycle and suggested that embedding treatment-free intervals (TFIs) in this phase can improve TCE responses.^6^ In both *in vitro* and *in vivo* models using blinatumomab, intermittent dosing regimens that included 7-day TFIs led to improved tumor rejection compared to continuous administration. As with most immunotherapies, treatment failure in TCE therapy is often marked by increased T cell exhaustion, underscoring the importance of strategies that can mitigate this dysfunction.^7^ Thus, TFI-based regimens represent a promising approach to improve response durability and overcome resistance.

While TFI-based administration improved TCE efficacy over continuous dosing in the study by Philipp et al., the selection of a 7-day interval appears empirically chosen rather than biologically optimized. To systematically explore alternative TFI schedules and identify regimens that reduce T cell exhaustion while maintaining antitumor activity, we calibrated an agent-based model (ABM) using the *in vitro* data reported by Philipp et al.^6^ ABMs have been widely used in cancer systems modeling to investigate immunotherapy, resistance mechanisms, and treatment scheduling. ^8,9^ ABMs simulate individual cell behaviors and their interactions within a spatially explicit microenvironment,^10^ allowing for stochastic variation, spatial constraints, and rule-based dynamics that more closely reflect tumor–immune interactions observed *in vivo*.^10^ By incorporating biological rules governing T cell activation, proliferation, exhaustion, and cytotoxicity, our ABM provides a virtual testbed for evaluating how different TFI structures influence therapeutic outcomes, enabling hypothesis generation and prioritization of experimental strategies that would be impractical to test exhaustively in preclinical models.

## Methods

Mathematical modeling has long been recognized as a powerful and efficient approach for testing and predicting treatment strategies in cancer.^11^ Among these approaches, ABM represents a particularly versatile computational framework in which individual entities, referred to as *agents*, follow predefined behavioral rules and interact with each other and their environment. By simulating these local interactions, ABMs enable the emergence of complex, system-level behaviors that cannot be inferred directly from individual components. These models are inherently stochastic and constructed from the bottom up, assigning distinct properties and behavioral rules to each agent and allowing dynamic interactions that collectively give rise to emergent phenomena. The use of ABMs in cancer research is well established and has proven valuable for integrating biological complexity across scales and disciplines.^12^

### Agent based model of tumor- T cell interactions

**Figure 1:**
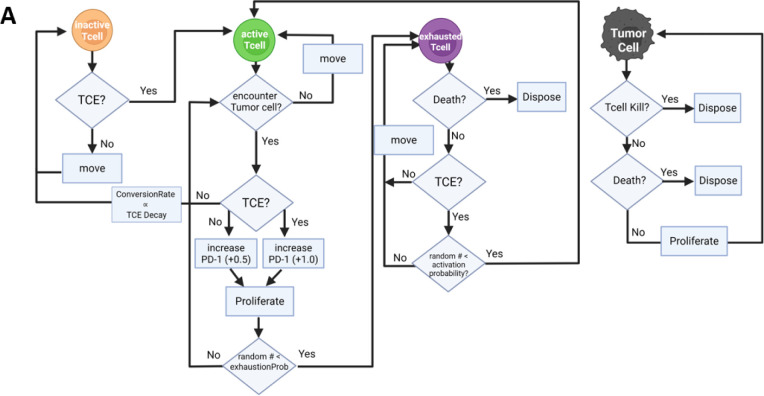

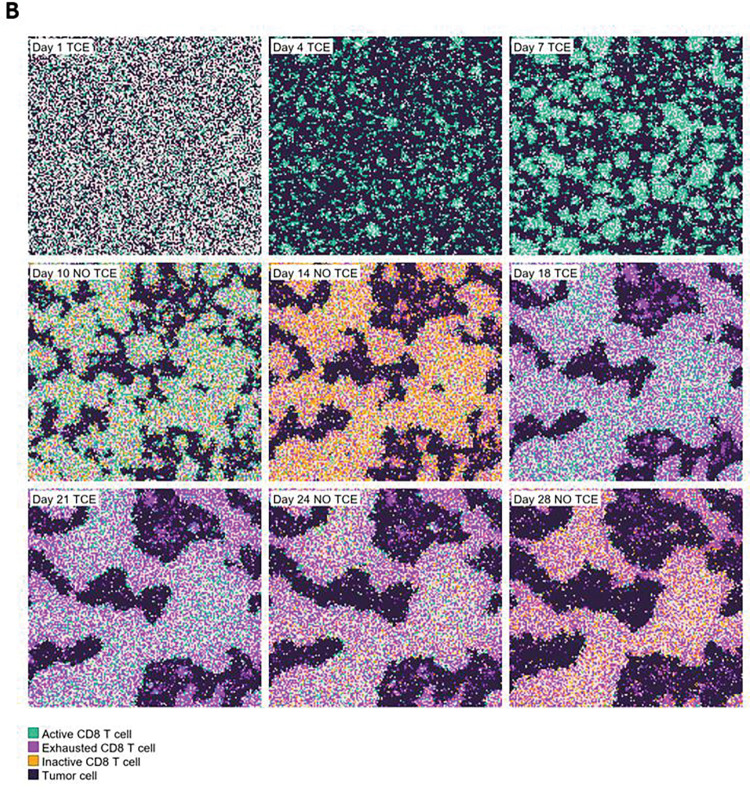
Agent decision logic and spatial dynamics of ABM. (A) Decision tree illustrating the rules governing state transitions and behaviors of inactive, active, and exhausted CD8^+^ T cells, and tumor cells, in response to TCE therapy. Transitions depend on TCE exposure, tumor encounters, PD-1 accumulation, and stochastic events controlling activation, proliferation, exhaustion, reversion, and death. (B) Representative spatial snapshots from a single ABM replicate. Each panel shows one simulation day; frames are sampled at ~3–4-day intervals. Panels are annotated with the simulation day and treatment status (TCE when therapy is on; NO TCE otherwise). In this run, TCE was administered on days 1–7 and 15–21. Colors: active CD8^+^ T cells (teal), exhausted CD8^+^ T cells (purple), inactive CD8^+^ T cells (orange), tumor cells (black).

The ABM was developed using the Hybrid Cellular Automata (HCA) framework, which has been widely applied to investigate cellular interactions and evolutionary dynamics in diverse biological systems.^13^ The HCA paradigm represents discrete agents that follow a defined set of behavioral rules within a spatially explicit environment, updated at each time step according to flowcharts describing their biological processes (Figure 1A). The model was implemented using the Hybrid Automata Library (HAL).^14^

In this framework, each agent corresponds to an individual cell, and agents are of two possible types: tumor cells and T cells. All interactions are governed by direct cell–cell contact and local spatial organization. Agents reside on a two-dimensional lattice (150 × 160 pixels), representing a subsection of a petri dish well where tumor cells and T cells are co-cultured, as described by Philipp et al.^6^ Each lattice site can contain at most one cell, and each time step corresponds to one hour of real time.

Agents interact through a Moore neighborhood, which includes the eight surrounding lattice sites in the horizontal, vertical, and diagonal directions. This spatial configuration defines the set of possible interactions such as cell movement, immune killing, and tumor proliferation. The Moore neighborhood captures the localized nature of cell–cell interactions observed in co-culture systems, where physical proximity governs the likelihood of contact-mediated processes.

### Model Initialization and Agent Rules

Initial conditions were informed by the *in vitro* co-culture experiments reported by Philipp et al.^6^ The model was initialized at an effector-to-target (E:T) ratio of 1:4, corresponding to 20% T cells and 80% tumor cells. To emulate typical experimental conditions with partial confluency, the overall occupancy of the grid was set to 50%, allowing cells to move and interact dynamically. Given a 150 × 160 grid (24,000 pixels total), this corresponds to 12,000 occupied sites, initialized with 2,400 T cells and 9,600 tumor cells (Figure 1B).

All cells were placed randomly on the grid at the start of the simulation. Each agent updates sequentially according to stochastic rules governing its behavior, which include proliferation, death, movement, and, for T cells, the ability to kill adjacent tumor cells. The probability and timing of these events are defined by experimentally informed parameters (Table 1). The interactions between tumor and T cells within this spatial structure enable the emergence of heterogeneous local dynamics resembling those observed *in vitro*.

### Tumor Cell Behavior

Tumor cells represent pre-irradiated CD19^+^ lymphoma cells and are initialized as non-motile agents randomly distributed on the grid. At each 1-hour time step, each tumor cell (i) undergoes a stochastic apoptosis check (per-hour probability 1/72); cells that die are removed from the lattice, and (ii) if alive, may proliferate with probability 1/24, placing a daughter into a neighboring empty site. Independently, tumor cells can be eliminated by T cell-mediated cytotoxicity when encountered by active T cells.

### T cell Behavior

T cells are modeled as individual agents capable of movement, cytotoxicity (killing tumor cells), proliferation, exhaustion, and death. Each T cell can have one of three functional states—inactive active, or exhausted—and can transition between these states based on interactions with the tumor microenvironment.

Inactive T cells represented those whose T cell receptors (TCRs) are not tumor-specific and therefore unable to kill tumor cells. Only active T cells kill tumor cells or undergo proliferation. T cells move stochastically at each time step (1 hour) into nearby unoccupied grid spaces.

Active T cells perform up to three Moore-neighborhood scans per hour; if no target is engaged, they move stochastically into a random empty neighboring site. Upon encountering a tumor cell, an active T cells attempts to kill it according to a probabilistic rule. Following a successful kill, the active T cells became eligible to divide, provided at least 5 hours had passed since its last division.^18^ T cell proliferation is inversely proportional to the total number of T cells on the grid, representing competition for limiting resources such as IL-2.^21^ After tumor cell killing, active T cells accumulate PD-1 expression; once the PD-1 level exceeds a defined exhaustion threshold, the active T cell becomes exhausted, and is incapable of killing and could undergo cell death.

Exhausted T cells were assigned a probabilistic death rate calibrated to published *in vitro* survival data, which showed that CD8^+^ T cells stimulated in IL-2 undergo approximately 40% cell death within 10 days of activation. Based on this, we set the hourly death probability of exhausted T cells using an exponential decay function, setting the rate to 0.00213 per hour so that ~40% of exhausted T cells would die within 240 hours (10 days), consistent with the reported survival curve.^22^

When TCE was present, there was a low probability (“activation probability”) that exhausted T cells could be converted back to active T cells. Since the study by Philipp et al. used T cells isolated from peripheral blood (PB) of healthy donors, we assumed that most T cells to be inactive at model initialization. No T cells were initialized as exhausted, consistent with the very low PD-1 expression typically observed on T cells in peripheral blood, especially of healthy individuals.^23,24^

### Effect of T cell engager (TCE) therapy

The presence of TCE was approximated with a step function so that treatment is either on or off. In the former case all cells are exposed to drug using a Boolean flag (TCE_ON) that can be toggled on or off. This allowed the simulation to incorporate treatment-free intervals (TFIs) and evaluate the impact of intermittent dosing.

When TCE treatment is on, inactive T cells convert into active T cells. This reflects the mechanism of action of bispecific antibodies such as blinatumomab, which engage CD3 (a universal T cell marker) and redirect T cells toward tumor-associated antigens, bypassing the physiological requirement for TCR–MHC recognition. TCEs, by comparison, deliver potent, non-physiological activation signals that bypass these checkpoints. This supraphysiological stimulation, driven by high-affinity CD3 engagement, enables rapid tumor killing but also accelerates T cell exhaustion. In fact, recent efforts in TCE design have focused on reducing CD3 binding affinity to mitigate exhaustion while preserving antitumor efficacy.^25^

Accordingly, PD-1 accumulation is modeled to occur at a higher rate during tumor cell killing events that took place under TCE exposure. Once PD-1 expression exceeded a defined exhaustion threshold, active T cells transitioned to an exhausted state, with reduced cytotoxic and proliferative function.

This modeling approach reflects a key biological trade-off: while TCEs can rapidly mobilize a broad population of T cells to mediate antitumor effects, they do so at the cost of sustained functionality. By incorporating these assumptions, the model enables systematic exploration of alternative TCE dosing schedules to balance early tumor clearance with long-term T cell persistence.

In the experimental system we modeled, the half-life–extended CD19×CD3 bispecific molecule AMG 562 (half-life ~210 hours) was used in place of blinatumomab, a first-generation CD19×CD3 TCE with a much shorter half-life (~2 hours). To reflect the pharmacokinetics of AMG 562 and the rationale behind daily TCE administration, we assumed that T cells could revert from active to inactive, specifically, when TCE is turned off, active T cells begin to accumulate “hours since activation” and become eligible to revert to an inactive state based on an exponential decay function that mimics TCE clearance. The probability of reversion increases over time and follows a standard half-life decay curve (e.g., 50% chance after 210 hours, 75% after 420 hours). This approach simulates the gradual loss of TCE signaling following treatment cessation. Importantly, PD-1 expression is preserved during reversion, capturing the persistence of exhaustion-related signaling. This mechanism allows the model to represent both the pharmacologic decline of TCE and the potential for T cell dormancy between treatment intervals.

### Data Analysis, Visualization, and Reproducibility

To account for the model’s stochastic nature, each condition was simulated across 50 independent iterations. This repetition allowed assessment of outcome variability and robustness. A formal power analysis was not performed, given the ability to generate large synthetic datasets; instead, reproducibility was ensured by initializing each run with a fixed random seed.

Statistical comparisons between two groups were performed using Welch’s t-test. For comparisons involving multiple groups, p-values were adjusted using the Bonferroni correction method. The full simulation code is available at: https://github.com/ninaobertopp/TFI

## Results

### Mathematical Model Reproduces *In vitro* Benefits of Intermittent TCE Therapy

**Figure 2:**
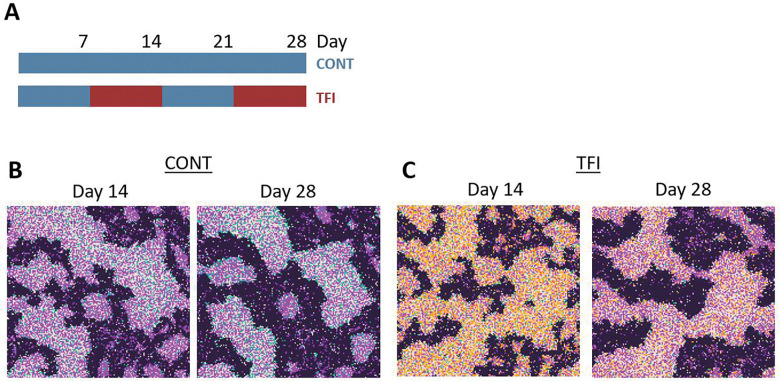

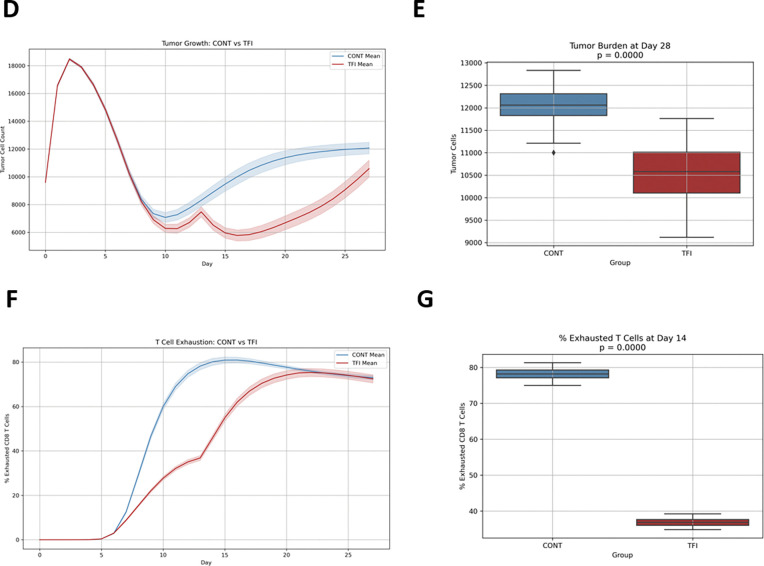
Simulations recapitulating preclinical CD8^+^ T cell dynamics during the initial 28-day TCE treatment phase. (A) Visualization of the two TCE treatment schedules simulated over 28 days. Top: Continuous TCE treatment (CONT), with TCE administered from Day 1 to Day 28 (blue). Bottom: Treatment-free interval (TFI) schedule, with TCE administered during Weeks 1 and 3 (blue) and withdrawn during Weeks 2 and 4 (red). (B+C) Simulated spatial distributions of tumor cells and T cells under (B) CONT and (C) TFI therapy. Black pixels represent tumor cells; colored pixels represent T cells in distinct functional states: inactive (orange), active (green), and exhausted (purple). (D) Simulated tumor growth under CONT versus TFI therapy, showing total tumor cell counts over time (mean ± SD). (E) Tumor burden at Day 28 under CONT and TFI conditions. Each boxplot displays the distribution of total tumor cell counts. (F) Temporal dynamics of CD8^+^ T cell exhaustion under CONT and TFI, shown as the percentage of exhausted T cells over time (mean ± SD). (G) CD8^+^ T cell exhaustion at Day 14 under CONT and TFI. Boxplots show the percentage of exhausted T cells. All data represent n = 50 simulation replicates per group. Statistical comparisons were performed using Welch’s t-test.

Previously, an *in vitro* lymphoma co-culture model of TCE therapy compared to a continuous 28-day administration of blinatumomab (CONT), reflecting current clinical practice, to an intermittent regimen incorporating 7-day treatment-free intervals (TFI).^6^ The study assessed T cell proliferation and cytotoxicity (tumor cell killing) on days 14 and 28 and found that T cells cultured under TFI conditions exhibited increased proliferation and cytotoxicity, along with reduced exhaustion, compared to those under CONT conditions. To evaluate whether our ABM, could reproduce these experimental findings, we simulated CONT and TFI conditions over a 28-day period (Figure 2 A-C).

The ABM successfully recapitulated key experimental outcomes. At Days 14 and 28, TFI yielded higher numbers of active T cells, less proportion of exhausted T cells and fewer tumor cells than CONT, consistent with enhanced proliferation and cytotoxic activity (Fig. 2B–C). Despite both schedules initially reducing tumor burden, tumor regrowth was more pronounced under the continuous schedule, resulting in significantly higher tumor cell counts by Day 28 (Fig. 2D–E).

Notably, T cell exhaustion dynamics diverged sharply between regimens. CONT triggered a rapid rise in CD8^+^ T cell exhaustion, exceeding 80% by Day 16. In contrast, the TFI schedule substantially delayed and limited exhaustion, with a lower peak and slower rate of accumulation (Fig. 2F). At Day 14, the proportion of exhausted T cells was significantly lower under TFI compared to CONT (Welch’s t-test, *p* < 0.0001), reflecting an early protective effect of the intermittent regimen (Fig. 2G).

Together, these results confirm that the ABM captures key functional advantages of intermittent TCE therapy observed *in vitro*, validating the model’s use for subsequent exploration of alternative dosing strategies.

### Loss of 7-Day TFI Superiority Over a Full Treatment Cycle

**Figure 3:**
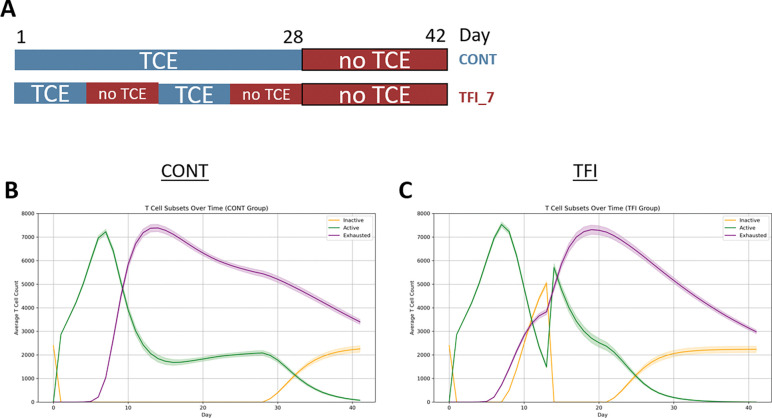

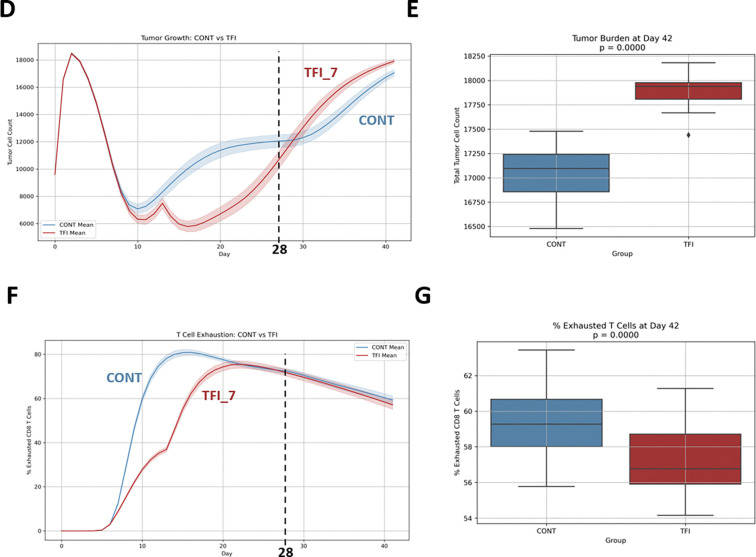
Full-cycle simulation (42 days) with 14-day treatment-free phase. (A) Visualization of the two TCE treatment schedules simulated over 42 days. Top: Continuous TCE treatment (CONT), with TCE administered from Day 1 to Day 28 (blue), followed by a 14-day treatment-free period (red). Bottom: Treatment-free interval (TFI) schedule, with TCE administered during Weeks 1 and 3 (blue) and withdrawn during Weeks 2 and 4 (red), followed by a 14-day treatment-free period (red). (B+C) Dynamics of CD8^+^ T cell functional subsets under CONT (B) and TFI (C) treatment. Plots show the mean ± standard deviation of inactive (orange), active (green), and exhausted (purple) T cell counts. (D) Simulated tumor growth under CONT versus TFI therapy, showing total tumor cell counts over time (mean ± SD). (E) Tumor burden at Day 42 under CONT and TFI conditions. Each boxplot displays the distribution of total tumor cell counts. Welch’s t-test (p < 0.0001). (F) Temporal dynamics of CD8^+^ T cell exhaustion under CONT and TFI, shown as the percentage of exhausted T cells over time (mean ± SD). (G) CD8^+^ T cell exhaustion at Day 42 under CONT and TFI. Boxplots show the percentage of exhausted T cells. Welch’s t-test (p < 0.0001). All data represent n = 50 simulation replicates per group. Statistical comparisons were performed using Welch’s t-test.

While the *in vitro* study by Philipp et al. evaluated TCE therapy over a single 28-day administration period, clinical protocols typically include a 14-day treatment-free phase following each 28-day cycle of blinatumomab infusion.^4^ To better reflect this clinical reality, we extended our ABM simulations to cover the full 42-day cycle, including both treatment and rest phases (Fig. 3A).

Under continuous treatment (CONT), active CD8^+^ T cells expanded rapidly during the first week, peaking around Day 8, and were subsequently overtaken by the exhausted population by Day 10 (Fig. 3B). While exhaustion became the dominant state, a moderate number of active T cells persisted throughout the treatment phase. Inactive T cells began to reaccumulate only after Day 28, coinciding with treatment withdrawal. In contrast, the intermittent schedule (TFI) maintained higher active T cell levels for longer, with visible rebounds in the inactive population during each 7-day treatment-free interval (Fig. 3C). These periods of withdrawal also coincided with slower exhaustion buildup and transient reductions in the exhausted T cell fraction, suggesting that intermittent dosing temporarily interrupts exhaustion trajectories and supports partial T cell recovery.

However, despite the initial advantages observed under TFI, extending the simulation to Day 42 revealed a reversal in treatment efficacy. By the end of the full cycle, CONT yielded significantly lower tumor burden than TFI (Fig. 3D–E), challenging the prior conclusion that intermittent dosing is consistently superior. Although TFI still limited exhaustion more effectively (Fig. 3F–G), this benefit did not translate into superior tumor control at the end of the full treatment cycle.

This apparent disconnect likely reflects a trade-off between preserving T cell functionality and maintaining cumulative cytotoxic pressure on the tumor. While intermittent dosing mitigates T cell exhaustion, it also interrupts tumor killing during treatment-free intervals. As a result, tumors are afforded time to regrow during these off periods, and many T cells revert to an inactive state, diminishing overall cytotoxic activity. In contrast, continuous TCE exposure sustains active killing, even at the cost of exhaustion, which may lead to more consistent tumor suppression over time. Thus, while reduced exhaustion under TFI is immunologically favorable, it alone is insufficient to ensure superior tumor control if cumulative tumor clearance is compromised.

Given these findings, we next asked whether a modified TFI regimen with shorter treatment-free intervals might strike a better balance between preserving T cell functionality and limiting tumor regrowth.

### Optimizing Intermittent Dosing: Shorter TFIs Outperform 7-Day and Continuous TCE Schedules

**Figure 4:**
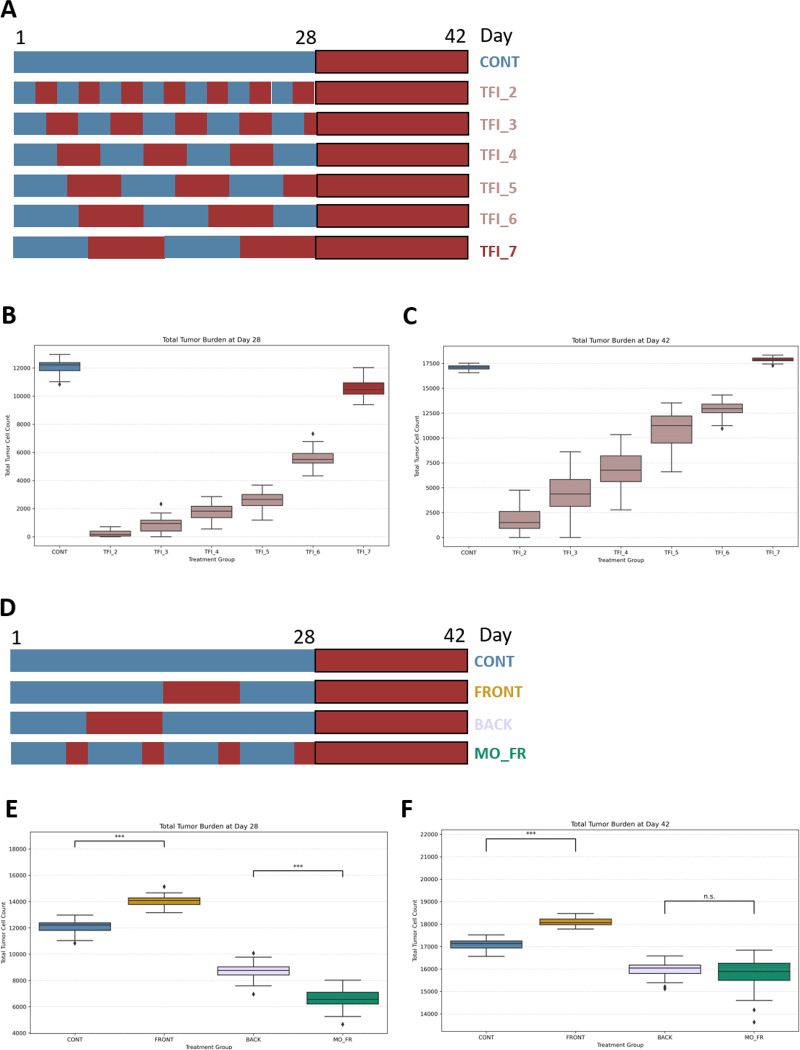
Impact of Intermittent and Translationally Oriented TCE Treatment Schedules on Tumor Burden (A) Schematic overview of TCE administration schedules simulated over 42 days. In the continuous treatment group (CONT), TCE is delivered daily from Day 1 to Day 28 (blue), followed by a 14-day treatment-free period (red). Intermittent schedules TFI_2 through TFI_7 incorporate recurring treatment-free intervals (2–7 days, respectively), alternating with periods of TCE dosing during the initial 28 days, followed by a uniform 14-day treatment-free phase from Day 29 to 42. Black borders indicate the start of the treatment-free period. (B) Total tumor burden on Day 28 across continuous and intermittent TCE regimens. Boxplots show tumor cell counts for CONT and each TFI group. (C) Tumor burden at Day 42 following continuous or intermittent TCE treatment. (D) Overview of translationally oriented TCE schedules. Front-loaded (FRONT): TCE is administered during the first 14 days, followed by a 7-day break and a final treatment week. Back-loaded (BACK): TCE is administered during the first 7 days, followed by a 7-day break and then 14 consecutive days of treatment. Monday through Friday (MO_FR): TCE is administered only on weekdays with no dosing on weekends. All groups transition to a uniform 14-day treatment-free phase from Day 29 to Day 42. (E) Tumor burden at Day 28 under translationally oriented TCE schedules, comparing outcomes across CONT, FRONT, BACK, and MO_FR groups. (F) Tumor burden at Day 42 under the same regimens, assessing durability of tumor control. Statistical comparisons were conducted using Welch’s t-test with Bonferroni correction. All group differences were significant at ***P < 0.0001 unless otherwise indicated; n = 50 per group.

We next used our calibrated ABM to explore alternative TCE dosing strategies beyond the originally proposed 7-day TFI regimen. We simulated a range of intermittent schedules (TFI_2 through TFI_7) that varied the length of treatment-free intervals from 2 to 7 days, while maintaining a consistent 28-day treatment window followed by a 14-day resting phase (Fig. 4A). Our goal was to identify whether shorter, more frequent interruptions in therapy could improve tumor control by better balancing cytotoxic efficacy and T cell preservation.

At Day 28, shorter TFIs demonstrated a clear advantage: tumor burden increased progressively with longer treatment-free intervals, with TFI_2 and TFI_3 achieving the greatest tumor reduction (Fig. 4B). As previously shown, the 7-day TFI regimen initially outperformed continuous treatment (CONT) at this timepoint, but this benefit did not persist through the full 42-day cycle. In contrast, all shorter TFI schedules (2–6 days) maintained significantly lower tumor burden than both CONT and TFI_7 at both timepoints. Notably, tumor control improved in a stepwise manner as treatment-free intervals shortened. These findings indicate that shorter, more frequent interruptions not only preserve early efficacy but also sustain therapeutic benefit throughout the full treatment cycle (Fig. 4C).

To explore how intermittent dosing could be translated into clinically more feasible regimens, we next simulated front-loaded (FRONT), back-loaded (BACK), and weekday-only (MO_FR) schedules (Fig. 4D). At Day 28, the MO_FR schedule achieved the lowest tumor burden, outperforming both FRONT and BACK regimens (Fig. 4E). However, by Day 42, the difference between MO_FR and BACK was no longer statistically significant, suggesting that although MO_FR may offer early benefit, back-loaded therapy may elicit a delayed but ultimately comparable effect (Fig. 4F). In contrast, the FRONT-loaded regimen consistently underperformed, resulting in higher tumor burden than continuous dosing at both timepoints.

Together, these results emphasize the importance of both interval length and timing in optimizing TCE therapy.

## Discussion

This study demonstrates the utility of ABMs in refining TCE therapy, specifically through systematic exploration of TFIs. While the 7-day TFI regimen initially appeared superior to continuous treatment in short-term experiments, our simulations reveal that this advantage does not hold when the full 42-day clinical cycle is considered. Instead, shorter TFIs provided more durable tumor control while limiting T cell exhaustion, outperforming both 7-day TFI and continuous treatment across all timepoints.

These findings suggest that brief, frequent interruptions in TCE therapy may better balance sustained cytotoxic pressure with preservation of T cell functionality. Importantly, tumor control improved progressively as the length of treatment-free intervals decreased. Based on our results, shorter TFIs should be considered as an approach for enhancing TCE efficacy—provided they are clinically feasible.

However, implementing very short TFIs in clinical practice may pose practical challenges for both healthcare providers and patients. To bridge this gap between mechanistic optimization and real-world feasibility, we evaluated regimens that are translationally more relevant such as the Monday-through-Friday (MO_FR) schedule. While slightly less effective than short TFIs, the MO_FR schedule consistently outperforms continuous treatment and provides a practical compromise between therapeutic benefit and clinical feasibility.

An important nuance is that the loss of 7-day TFI superiority at the day-21 timepoint, may be driven by the additional 14-day inter-cycle rest that separates 4-weeks cycles of continuous treatment. In other words, a 7-day intra-cycle TFI plus a 14-day inter-cycle treatment holiday compounds off-drug time, permitting increased T cell disengagement and tumor regrowth. If 7-day TFIs are retained for operational feasibility, our results motivate reexamining the length of the subsequent break between cycles. Shortening or omitting the standard 14-day inter-cycle pause following 7-day TFI may preserve antitumor control while limiting T cell exhaustion.

This study highlights the power of mathematical models to not only reproduce experimental findings but also evaluate a broad range of treatment schedules that would be prohibitively time consuming and resource intensive to test experimentally. Our simulations identified the 2-day and 3-day TFI regimens, as well as the MO_FR schedule, as particularly effective in reducing tumor burden and limiting T cell exhaustion.

It is also important to acknowledge the limitations of our current model. While the ABM was calibrated using experimental data from Philipp et al.^6^, the underlying system represents an *in vitro* co-culture environment. In those experiments, the clinically used blinatumomab, which has a short half-life of approximately 2 hours, was replaced with AMG 562, a CD19xCD3 bispecific molecule with an extended half-life of about 210 hours. To reflect this in our model, we incorporated a probabilistic decay function based on the reported half-life of AMG 562. This allowed us to simulate a gradual decline in engager activity and capture T cell reversion dynamics during treatment-free intervals. However, it is important to note that pharmacokinetic properties such as clearance and half-life do not translate directly between *in vitro* and *in vivo* systems, since *in vitro* models lack metabolic and elimination processes.^26^ As a result, the actual half-life of AMG 562 in the *in vitro* setting may be longer than modeled.

In addition to these pharmacokinetic simplifications, our ABM incorporates several biological assumptions that may not fully reflect the complexity of tumor–immune interactions. For example, we made parametric assumptions such as the rate of PD-1 accumulation during TCE-driven killing, the probability of successful tumor cell killing, and the required resting period before T cell division. The model also includes important non-parametric assumptions, including the fixed size and shape of the spatial grid, and the decision to randomly seed tumor and T cells across the grid rather than modeling spatial clustering. To ensure the robustness of our conclusions, future work will include systematic sensitivity analysis to evaluate how both parametric and non-parametric elements affect simulation outcomes, identify the most influential factors, and determine whether key predictions hold across a biologically plausible range of conditions.^27^

Additionally, we aim to adapt our model to *in vivo* systems, where the biological context is more complex and the pharmacokinetics of blinatumomab can be more accurately represented. The much shorter half-life of blinatumomab is expected to cause a faster and more pronounced decline in TCE activity during treatment pauses, which could amplify the differences between continuous and intermittent dosing. Incorporating these pharmacodynamic features, along with more realistic tumor–immune interactions and immunosuppressive mechanisms, will allow for more accurate prediction of treatment outcomes under clinically relevant conditions. Nonetheless, the candidate regimens identified by the ABM will require experimental validation through follow-up *in vitro* studies and, ultimately, *in vivo* testing to confirm their biological relevance and translational potential. Ultimately, this integrated modeling and experimental framework will support the design of clinical trials aimed at evaluating intermittent TCE regimens that improve efficacy, limit exhaustion, and enhance treatment durability.

## Figures and Tables

**Table 1:** Model parameters and sensitivity ranges.

Parameter	Symbol	Baseline value	Range / Values Tested	Unit	Notes / Rationale
Grid size	–	150 × 160	fixed	pixels	2D subsection of culture well (24,000 sites).
Grid occupancy	–	0.5	fixed	fraction	50% confluency replicating *in vitro* setup.^6^
Initial E:T ratio	E:T	1:4	fixed	ratio	20% T cells, 80% tumor cells.^6^
Time step	Δt	1	fixed	hour	Each step ≈ 1 hour.
Tumor proliferation	p_prolif	1/24	1/24 – 1/48	h^−1^	Matches daily lymphoma cell division.^15^
Tumor apoptosis	p_death	1/132	1/72 – 1/200	h^−1^	Low background apoptosis.^16^
Tumor motility	–	0	fixed	–	Tumor cells non-motile (pre-irradiated).
Inactive T cells	–	100% initial	fixed	–	Consistent with PBMC activation state.
Active T cells	–	0	fixed	–	Activated by TCE.
Exhausted T cells	–	0	fixed	–	Arise after PD-1 threshold exceeded.
T cell movement	–	1	fixed	step/hour	Random walk into empty site.
Killing probability	p_kill	0.03	0.02 – 0.06	per contact	Calibrated to CD19xCD3 killing kinetics.^17^
Division cooldown	τ_div	5	5–28	hours	Minimum time between divisions.^18^
Division scale	s_div	0.25	0.15 – 0.35	–	Captures IL-2 competition dynamics.
PD-1 increase per kill	ΔPD1	0.35	0.2 – 0.45	a.u./kill	Higher accumulation under TCE.
Exhaustion threshold	θ_exh	13	12 – 14	a.u.	Threshold for exhausted state.
Exhausted death rate	p_death_exh	0.00213	fixed	h^−1^	~40% death by 10 days.^19^
TCE reversion t_½_	t½_TCE	210	150 – 300	hours	According to AMG-562 PK.^20^
ActiveX→inactive reversion	p_rev(t)	exp-decay	fixed	–	Half-life driven loss of signal.

## References

[1] National Cancer Institute (NCI) – SEER Program. SEER Cancer Stat Facts: Acute Lymphocytic Leukemia. Available at https://seer.cancer.gov/statfacts/html/alyl.html (2025).

[2] Testa U., Pelosi E., Castelli G. & Chiusolo P. Blinatumomab in the Therapy of Acute B-Lymphoid Leukemia. Mediterranean journal of hematology and infectious diseases 16, e2024070; 10.4084/MJHID.2024.070 (2024). 39258182 PMC11385599

[3] Ahmed Ibrahim MD A Systematic Review on Efficacy and Safety Profile of Blinatumomab in Relapsed or Refractory B-Cell Precursor Acute Lymphoblastic Leukemia (R/R BCP-ALL) in Children and Young Adults. Blood 144, 5888 (2024).

[4] BLINCYTO^®^ (blinatumomab) Dosing. Available at https://www.blincytohcp.com/dosing (2025).

[5] U.S. Food and Drug Administration, Center for Drug Evaluation and Research. Clinical Pharmacology and Biopharmaceutics Review: BLINCYTO (blinatumomab), BLA 125557 (2014).

[6] Philipp N. T-cell exhaustion induced by continuous bispecific molecule exposure is ameliorated by treatment-free intervals. Blood 140, 1104–1118; 10.1182/blood.2022015956 (2022). 35878001 PMC10652962

[7] Friedrich M. J. The pre-existing T cell landscape determines the response to bispecific T cell engagers in multiple myeloma patients. Cancer cell 41, 711–725.e6; 10.1016/j.ccell.2023.02.008 (2023). 36898378

[8] Bekker R. A. Spatially fractionated GRID radiation potentiates immune-mediated tumor control. Radiation Oncology (London, England) 19, 121; 10.1186/s13014-024-02514-6 (2024). 39272128 PMC11401399

[9] Valentinuzzi D. & Jeraj R. Computational modelling of modern cancer immunotherapy. Physics in medicine and biology 65, 24TR01; 10.1088/1361-6560/abc3fc (2020). 33091898

[10] Wang Y. Agent-Based Modeling of Virtual Tumors Reveals the Critical Influence of Microenvironmental Complexity on Immunotherapy Efficacy. Cancers 16, 2942; 10.3390/cancers16172942 (2024). 39272799 PMC11394213

[11] Altrock P. M., Liu L. L. & Michor F. The mathematics of cancer: integrating quantitative models. Nature reviews. Cancer 15, 730–745; 10.1038/nrc4029 (2015). 26597528

[12] West J., Robertson-Tessi M. & Anderson A. R. A. Agent-based methods facilitate integrative science in cancer. Trends in cell biology 33, 300–311; 10.1016/j.tcb.2022.10.006 (2023). 36404257 PMC10918696

[13] Anderson A. R. A. A hybrid mathematical model of solid tumour invasion: the importance of cell adhesion. Mathematical medicine and biology : a journal of the IMA 22, 163–186; 10.1093/imammb/dqi005 (2005). 15781426

[14] Bravo R. R. Hybrid Automata Library: A flexible platform for hybrid modeling with real-time visualization. PLOS Computational Biology 16, e1007635; 10.1371/journal.pcbi.1007635 (2020). 32155140 PMC7105119

[15] Molyneux E. M. Burkitt’s lymphoma. Lancet (London, England) 379, 1234–1244; 10.1016/S0140-6736(11)61177-X (2012). 22333947

[16] Witkowska M. Spontaneous in vitro apoptosis of de novo chronic lymphocytic leukemia cells correlates with risk of the disease progression. Cytometry Part B: Clinical Cytometry 86, 410–417; 10.1002/cyto.b.21163 (2014). 24515875

[17] Hoffmann P. Serial killing of tumor cells by cytotoxic T cells redirected with a CD19-/CD3-bispecific single-chain antibody construct. International journal of cancer 115, 98–104; 10.1002/ijc.20908 (2005). 15688411

[18] Plambeck M. Heritable changes in division speed accompany the diversification of single T cell fate. Proceedings of the National Academy of Sciences of the United States of America 119; 10.1073/pnas.2116260119 (2022). PMC8892279 35217611

[19] Yousif A. Faithful modeling of terminal CD8+T cell dysfunction and epigenetic stabilization in vitro. JCI Insight 10; 10.1172/jci.insight.191220 (2025). PMC12513483 41059569

[20] Amgen Inc. A Phase 1, First-in-Human, Open-Label Study Evaluating the Safety, Tolerability, Pharmacokinetics, and Efficacy of AMG 562 in Subjects With Relapsed/Refractory Diffuse Large B-Cell Lymphoma, Mantle Cell Lymphoma, or Follicular Lymphoma., Protocol 20170533; 07 Aug 2019.

[21] Feinerman O. Single-cell quantification of IL-2 response by effector and regulatory T cells reveals critical plasticity in immune response. Molecular systems biology 6, 437; 10.1038/msb.2010.90 (2010). 21119631 PMC3010113

[22] Hedfors I. A. & Brinchmann J. E. Long-term proliferation and survival of in vitro-activated T cells is dependent on Interleukin-2 receptor signalling but not on the high-affinity IL-2R. Scandinavian journal of immunology 58, 522–532; 10.1046/j.1365-3083.2003.01338.x (2003). 14629624

[23] Puntigam L. K. Immune Checkpoint Expression on Immune Cells of HNSCC Patients and Modulation by Chemo- and Immunotherapy. International journal of molecular sciences 21; 10.3390/ijms21155181 (2020). PMC7432918 32707816

[24] Zhu C. T cell exhaustion is associated with the risk of papillary thyroid carcinoma and can be a predictive and sensitive biomarker for diagnosis. Diagnostic pathology 16, 84; 10.1186/s13000-021-01139-7 (2021). 34465342 PMC8408957

[25] Sharon D. Bivalent BCMA Binding and Low Affinity CD3 T-Cell Engagement By Abbv-383 Drives Sustained Activation with Reduced T-Cell Exhaustion in Preclinical Models of Multiple Myeloma. Blood 142, 4666; 10.1182/blood-2023-179039 (2023).

[26] Benet L. Z. & Sodhi J. K. Can In Vitro-In Vivo Extrapolation Be Successful? Recognizing the Incorrect Clearance Assumptions. Clinical Pharmacology & Therapeutics 111, 1022–1035; 10.1002/cpt.2482 (2022). 34731496 PMC9542026

[27] Borgonovo E., Pangallo M., Rivkin J., Rizzo L. & Siggelkow N. Sensitivity analysis of agent-based models: a new protocol. Comput Math Organ Theory 28, 52–94; 10.1007/s10588-021-09358-5 (2022).

